# Stimulation of Baroresponsive Parts of the Nucleus of the Solitary Tract Produces Nitric Oxide-mediated Choroidal Vasodilation in Rat Eye

**DOI:** 10.3389/fnana.2016.00094

**Published:** 2016-10-07

**Authors:** Chunyan Li, Malinda E. C. Fitzgerald, Nobel Del Mar, Anton Reiner

**Affiliations:** ^1^Department of Anatomy and Neurobiology, The University of Tennessee Health Science CenterMemphis, TN, USA; ^2^Department of Ophthalmology, The University of Tennessee Health Science CenterMemphis, TN, USA; ^3^Department of Biology, Christian Brothers UniversityMemphis, TN, USA

**Keywords:** choroidal blood flow, superior salivatory nucleus, nucleus of solitary tract, autonomic, parasympathetic

## Abstract

Preganglionic parasympathetic neurons of the ventromedial part of the superior salivatory nucleus (SSN) mediate vasodilation of orbital and choroidal blood vessels, via their projection to the nitrergic pterygopalatine ganglion (PPG) neurons that innervate these vessels. We recently showed that the baroresponsive part of the nucleus of the solitary tract (NTS) innervates choroidal control parasympathetic preganglionic neurons of SSN in rats. As this projection provides a means by which blood pressure (BP) signals may modulate choroidal blood flow (ChBF), we investigated if activation of baroresponsive NTS evokes ChBF increases in rat eye, using Laser Doppler Flowmetry (LDF) to measure ChBF transclerally. We found that electrical activation of ipsilateral baroresponsive NTS and its efferent fiber pathway to choroidal SSN increased mean ChBF by about 40–80% above baseline, depending on current level. The ChBF responses obtained with stimulation of baroresponsive NTS were driven by increases in both choroidal blood volume (ChBVol; i.e., vasodilation) and choroidal blood velocity (ChBVel; possibly due to orbital vessel dilation). Stimulation of baroresponsive NTS, by contrast, yielded no significant mean increases in systemic arterial blood pressure (ABP). We further found that the increases in ChBF with NTS stimulation were significantly reduced by administration of the neuronal nitric oxide (NO) synthase inhibitor N^ω^-propyl-l-arginine (NPA), thus implicating nitrergic PPG terminals in the NTS-elicited ChBF increases. Our results show that the NTS neurons projecting to choroidal SSN do mediate increase in ChBF, and thus suggest a role of baroresponsive NTS in the BP-dependent regulation of ChBF.

## Introduction

The retina has two separate vascular supplies involved in its support: the retinal vessels within the retina itself, and the choroidal vessels external to Bruch’s membrane and the retinal pigment epithelium (Alm, 1992). The retinal vessels provide blood supply to the inner two-thirds of the retina, while the outer retina is avascular and nourished from the choroid (Alm, 1992). The choroid provides 65–85% of the oxygen to the retina, with variation among species (Alm and Bill, 1973; Alm, 1992). Diminishing choroidal blood flow (ChBF) by occluding one or more the posterior ciliary arteries feeding choroid (Loeffler et al., 1994; Hayreh, 2004) or occluding a vortex vein (Collier, 1967), rapidly destroys the outer retina, emphasizing the importance of ChBF for outer retinal health. Moreover, reductions in ChBF are observed in aging and various retinal diseases, such as systemic hypertension, diabetic retinopathy and age-related macular degeneration (Grunwald et al., 1998a,b, 2005; Fitzgerald et al., 2001; Ito et al., 2001; Pournaras et al., 2006; Metelitsina et al., 2008), raising the possibility that the ChBF reductions contribute to the retinal declines observed.

Although ChBF was once thought to respond passively to changes in perfusion pressure, recent studies have shown that ChBF remains stable over a systemic blood pressure (BP) range of about 35% above and below basal (Kiel and Shepherd, 1992; Lovasik et al., 2003; Reiner et al., 2003, 2011), with neural mechanisms apparently involved in the compensation (Kiel, 1999; Li et al., 2010, 2015; Reiner et al., 2010b). When BP is low, choroidal blood vessels dilate to maintain blood flow to the eye, and when BP is high, choroidal blood vessels constrict, preventing overperfusion, tissue edema, and oxidative injury. Neurogenic choroidal vasodilation is mediated by parasympathetic and sensory innervation of choroidal vessels, and neurogenic choroidal vasoconstriction is accomplished by sympathetic innervation. Some parts of the central circuitry subserving parasympathetic contributions to choroidal baroregulation have been characterized. In birds and mammals both, the parasympathetic pterygopalatine ganglion (PPG) innervates orbital vessels feeding the choroid, as well as choroidal vessels themselves (Stone et al., 1987; Cuthbertson et al., 1997, 2003; Jablonski et al., 2007; Reiner et al., 2012). In mammals, the choroid is supplied by the posterior and anterior ciliary arteries, arising from the ophthalmic artery, while in birds the choroidal arteries arising from the ophthalmotemporal artery supply the choroid (Cuthbertson et al., 1997; Morrison et al., 1999; Reiner et al., 2012). The PPG input to choroid and the choroidal feeder vessels employ the vasodilators nitric oxide (NO) and vasoactive intestinal polypeptide (VIP), and as a result the ophthalmic and posterior ciliary arteries in mammals and the choroidal and ophthalmotemporal arteries in birds, as well as choroidal vessels, are densely coated with nerve fibers containing neuronal NO synthase (which synthesizes NO in neurons) and VIP (Stone et al., 1987; Cuthbertson et al., 1997; Reiner et al., 2012). The preganglionic neurons regulating the PPG reside in the superior salivatory nucleus (SSN), the autonomic component of the facial motor complex in the hindbrain (Cuthbertson et al., 2003). In prior studies on the inputs to the SSN in rats, we found that a major input arose from the nucleus of the solitary tract (NTS) of the medulla (Li et al., 2010), and in more recent studies we have characterized the part of NTS from which this input arises (Li et al., 2015).

The NTS is organized into viscerotopic subdivisions that receive input selectively from the tongue, vasculature and thoracic and abdominal viscera (Loewy, 1990; Paton, 1999; Pilowsky and Goodchild, 2002). Cardiovascular afferents predominantly project to the ipsilateral interstitial and dorsal NTS subnuclei at and anterior to the level of the obex (Ciriello, 1983; Zhang and Ashwell, 2001). Our studies have demonstrated that the part of NTS receiving baroreceptor information lies within the region of NTS that projects directly to the ChBF-control neurons of SSN (Li et al., 2015). Since NTS input to SSN is excitatory (Agassandian et al., 2002, 2003; Li et al., 2015) and SSN activation increases ChBF (Steinle et al., 2000; Fitzgerald et al. unpublished observations), it seems likely that the NTS also exerts a vasodilatory effect on choroid. The present study directly examined the influence of NTS on the ipsilateral ChBF using Laser Doppler Flowmetry (LDF). We found that activation of the parts of NTS receiving baroreceptive input and/or responsive to BP signals increases ipsilateral ChBF, and that the peripheral PPG terminals mediating this effect appear to use NO as a vasodilator. These results are consistent with the view that the NTS is part of the circuit for the adaptive parasympathetic regulation of ChBF during systemic hypotension.

## Materials and Methods

### Experimental Overview

Adult male Sprague-Dawley (SD) rats (290–380 g; from Harlan, Indianapolis, IN, USA) were used in all present studies. Our overall goal was to identify the sites within NTS from which ChBF increases could be elicited, and relate them to the location of the subdivisions within NTS that are baroresponsive and project to prechoroidal SSN. To this end, we mapped the distribution within NTS of neurons that express c-fos upon systemic hypotension, and related this to the published terminal field within NTS of aortic and carotid sinus baroreceptor afferent input via the vagus and glossopharyngeal nerves (Ciriello, 1983; Housley et al., 1987; Altschuler et al., 1989), the distribution of neurons within NTS that project to prechoroidal SSN identified in our own prior work (Li et al., 2015), our own NTS stimulation mapping, and the published distribution of BP-responsive NTS sites (Rogers et al., 1993; Mayne et al., 1998). We used the systematic sampling of NTS sites with electrical stimulation while monitoring ChBF and systemic BP to map ChBF-responsive NTS sites. Histological analysis was used to reconstruct the stimulation sites. Additionally, in a separate set of rats, we examined the effect of neuronal nitric oxide synthase (nNOS) inhibition on ChBF responses to activation of NTS sites yielding ChBF increases, to assess the involvement of peripheral NO-mediated mechanisms in the parasympathetic choroidal vasodilation observed with activation of effective NTS sites. All experiments were in compliance with the ARVO statement on the Use of Animals in Ophthalmic and Vision Research, and with NIH and institutional guidelines.

### Systemic Hypotension and NTS Responses

Twenty-two adult male SD rats (280–440 g) were anesthetized for 90–150 min, using ip injection of 0.1 ml/100 g of a ketamine/xylazine mixture (87/13 mg/kg), with supplemental doses (0.05–0.07 ml of ketamine/xylazine mixture) every 25–30 min. The right femoral artery was cannulated for systemic arterial blood pressure (ABP) recording, and the right femoral vein for blood withdrawal. Body temperature was maintained at 37°C with a Harvard heating blanket and rectal thermoprobe. To prepare the surgical site, hair was removed by clippers from the ventrum of the hind legs. To create hypotension, 4–7 ml of blood was slowly withdrawn via the cannula in the right femoral vein. The hypotensive state was maintained for 40 to 160 min. Control rats were similarly prepared, but no blood withdrawn. After surgery, the rats were perfused with 4% paraformaldehyde prepared in 0.1 M sodium phosphate buffer (PB; pH 7.4) with 0.1 M lysine and 0.01 M sodium periodate (PLP fixative). The brains were removed, cryoprotected at 4°C, and sectioned frozen at 40 μm on a sliding microtome into eight separate series for each brain. Selected series of sections were incubated in rabbit-anti-c-fos diluted at 1:1000 (sc-52; Santa Cruz Biotechnology; Santa Cruz, CA, USA) overnight at room temperature. After rinsing, the sections were incubated in donkey-anti rabbit secondary antibody (1:200; Jackson ImmunoResearch Laboratories, Inc., West Grove, PA, USA) for 1 h and rabbit peroxidase-anti-peroxidase (Rb PAP; 1:1000; Jackson ImmunoResearch Laboratories, Inc., West Grove, PA, USA) for 1 h. The c-fos labeling was visualized using diaminobenzidine tetrahydrochloride (DAB), using a standard brown DAB reaction as in our prior studies (Li et al., 2010, 2015). The sections were then rinsed, mounted on gelatin-coated slides, air-dried, dehydrated and coverslipped with Permount^®^ (Fisher Scientific, Pittsburgh, PA, USA).

### Choroidal Blood Flow Surgical Preparation

Prior to surgery, 24 rats were anesthetized by ip injection of 0.1 ml/100 g of a ketamine/xylazine mixture (87/13 mg/kg), with supplemental doses (0.05–0.07 ml of ketamine/xylazine mixture) every 25–30 min. To prepare the surgical sites, hair was removed by clippers from the dorsum of the head and ventrum of the hind legs. Body temperature was maintained at 37°C with a Harvard heating blanket and rectal thermoprobe. To measure systemic ABP, the femoral artery was catheterized as follows. Segments of PE-50 polyethylene tubing that were 20–30 cm long were pre-soaked with heparinized saline (40 units/ml) for 24 h to prevent catheter-induced clot formation. Both femoral artery and vein were isolated from the surrounding connective tissue and cannulated using such tubing. A Digi-Med Blood Pressure Analyzer^TM^ (BPA-100, Micro-Med, Inc., Louisville, KY, USA) was used to measure ABP and heart rate (HR) via a pressure transducer connected to the femoral artery cannula. The femoral vein was cannulated for administration of N^ω^-propyl-l-arginine (NPA, 1–2 mg/kg, Tocris Bioscience), a highly selective NO synthase 1 (NOS1, i.e., neuronal NOS) inhibitor (Zhang et al., 1997; Cooper et al., 2000). The rats were then positioned in a stereotaxic device. A pulse oximeter on the tail was used to measure systemic blood oxygen saturation.

To prepare the eye for transcleral measurement of ChBF, fascia overlying the superior pole of the right eye and the Harderian gland were removed. The tip of a laser Doppler probe connected to a LASERFLO BPM^2^ blood perfusion monitor (Vasamedics; Eden Prairie, MN, USA or BPM 403A; TSI Incorporated., St. Paul, MN, USA) was positioned 1–3 mm above the sclera with a micromanipulator. Measurements of ChBF were made from the vascular bed beneath the sclera in the gap between the superior and medial rectus muscles, at the equator of the superior part of the eye. A small amount of Aquasonic ultrasound gel (Parker Laboratories, Inc., Fairfield, NJ, USA) or 33% glycerol—0.1 M sodium PB, was used in the interface between the probe tip and the sclera to prevent tissue drying during the experiment. For placement of a stimulating electrode, the skin over the skull was retracted and bone over NTS removed. The coordinates for NTS from the rat brain atlas of Paxinos and Watson (2009) were: −13.6 mm behind Bregma, −1.0 lateral to Bregma, and −5.2 below the brain surface. An insulated, stainless steel stimulating electrode (5MΩ; Catlog # 572000; A-M Systems Inc., Sequim, WA, USA) was then positioned stereotaxically in the right NTS. To minimize the damage to NTS during penetration, we also used glass micropipette electrodes for seven rats. Micropipettes were pulled from glass tubing (World Precision Instrument, 1B150F-4) with a PE-2 microelectrode puller (Narashige), and micropipettes with a diameter of 50–100 μm used for stimulation. Micropipettes were filled with artificial cerebral spinal fluid (ACSF) composed of (in mM): 126 NaCl, 26 NaHCO_3_, 3 KCl, 1.25 NaH_2_PO_4_, 2 MgCl_2_, 2 CaCl_2_, and 20 glucose; pH 7.3, 310 mOsm, and a platinum wire was inserted.

### Choroidal Blood Flow Measurement

ChBF was measured in the right eye using a laser Doppler flow probe connected to a LASERFLO BPM^2^ blood perfusion monitor. Our previous work has shown that ChBF can reliably be measured transclerally, using LDF (Fitzgerald et al., 1990, 1996, 2001, 2005; Zagvazdin et al., 1996b; Reiner et al., 2010b). The principles of LDF have been described in detail by others (Haumschild, 1986; Bonner and Nossal, 1990), and this approach provides an accepted relative index of ChBF, as well as relative indices of choroidal blood volume (ChBVol) and choroidal blood velocity (ChBVel), whose product is ChBF and which have been shown to linearly relate to ChBF as measured by LDF (Riva et al., 1994; Shepherd et al., 1997; Grunwald et al., 1998a; Chou et al., 2002; Gugleta et al., 2002; Garhöfer et al., 2005; Tonini et al., 2010; Xu et al., 2010; Falsini et al., 2011). Moreover, hemodilution studies in model systems have shown that blood flow as assessed by laser Doppler scales linearly with red blood cell count (Nilsson et al., 1980; Fischer et al., 1985; Almond and Wheatley, 1992). Nonetheless, studies comparing ChBVol and ChBVel values determined by LDF with choroidal red blood cell concentration and velocity determined by other means would be useful for better understanding the choroidal vascular dynamics suggested by LDF data. Although the LASERFLO BPM^2^ nominally presents ChBF values in millilitre blood per minute per 100 g tissue, ChBVol in 12,000 red blood cells per cubic millimeter of tissue, and ChBVel as 10 mm/s, because of uncertainties about how well choroidal tissue matches the assumptions of the algorithms used to derive these measures, ChBF is reported here as relative blood flow units (BFUs), ChBVol as relative blood volume units (BVolU), and ChBVel as relative blood velocity units (BVelU). The output of our LASERFLO BPM^2^ was imported to a ML880 PowerLab 16/30 data acquisition system (ADInstruments Inc., Colorado Springs, CO, USA), and the digitized data were collected and analyzed using LabChart v7 Pro software (ADInstruments Inc., Colorado Springs, CO, USA) installed on an iMac Apple computer. The data averaging window of the BPM^2^ was set at 0.3 s, with the sampling rate on PowerLab set at 200 samples/s.

### Choroidal Blood Flow: Stimulation of NTS

Microstimulation consisted of a 7 s train of 100 Hz, 0.5 ms duration anodal or cathodal current pulses delivered by a S48 stimulator (Grass Instrument Company, Quincy, MA, USA) and PSIU6 stimulus isolation unit (Grass Instrument Company, Quincy, MA, USA). The amplitude of the anodal current pulses was varied between 50–100 μA, while for cathodal current pulses were 20 μA. For these rats, we systematically sampled NTS sites at a series of penetrations that encompassed the rostral-caudal, medial-lateral, and dorsal-ventral extent of NTS. In our initial studies (*n* = 17), we used anodal stimulation as we had in prior ChBF studies (Fitzgerald et al., 1990, 1996, 2005). Because axons within 300–500 μm of the electrode tip are more readily stimulated than neuronal cell bodies by 50–100 μA anodal current pulses of 0.5 ms duration (Bagshaw and Evans, 1976), we found that anodal stimulation tended to identify the course taken by NTS axons en route to prechoroidal SSN, especially at the lower current levels. To better define the location of the perikarya within NTS driving ChBF increases, we used cathodal stimulation of NTS in systematic mapping studies in a second set of rats (*n* = 7), because it more effectively stimulates the axon hillock of perikarya (Ranck, 1975; Merrill et al., 2005; Lu et al., 2008), thereby providing better localization of the perikarya that drive ChBF increases from within NTS. Data presented here for each rat represent an average of 3–7 NTS stimulation trials. Data on ChBF, ChVol, ChBVel, and ABP are presented. For ChBF, ChVol, and ChVel, data are presented in arbitrary units, as in our prior studies (Fitzgerald et al., 2001, 2005; Reiner et al., 2003, 2010b, 2011).

### Inhibition of nNOS

In a separate set of eight rats prepared as described above for ChBF and ABP measurement and NTS stimulation, we studied the effects of nNOS inhibition on the ChBF increasing effects of NTS stimulation. In these studies, electrodes were placed in the right NTS, as confirmed by the evoked ChBF increases and subsequent histological evaluation of electrode placement. The effects of 100 μA current pulses on ChBF was determined prior to nNOS inhibition, in 3–7 stimulation trials. We then administered NPA (1–2 mg/kg, Tocris Bioscience) iv, and examined the effect on baseline ChBF and NTS-evoked increases in ChBF, ChBVol, ChBVel and arterial BP. After NPA administration, an additional 3–7 NTS stimulation trials were performed for these eight rats.

### Choroidal Blood Flow—Histological Analysis of Stimulation Sites

At the end of each ChBF recording session, rats were perfused transcardially with 150–200 ml sodium phosphate buffered saline (PBS, 0.85% sodium chloride dissolved in 0.01 M PB), followed by 400–500 ml fixative of 4% paraformaldehyde prepared in 0.1M sodium PB (pH7.4) with 0.1 M lysine and 0.01 M sodium periodate (the PLP fixative). Brains were removed and cryoprotected at 4°C for at least 24 h in 20% sucrose −10% glycerol −0.138% sodium azide in 0.1 M sodium PB. Brains were then frozen with dry ice, and coronal sections cut with a sliding microtome at 40 μm. Sections were collected as six parallel series. The free-floating sections were stored at 4°C in a 0.02% sodium azide/0.02% imidazole in 0.1 M PB solution until staining procedures were carried out.

We used three histological methods to confirm that the electrode placement was within NTS and led to NTS activation: (1) analysis of the location of the electrode tip in NTS in cresyl violet-stained sections; (2) analysis of NTS activation in c-fos immunostained sections; and (3) analysis of NTS activation in sections immunolabeled for both parvalbumin and c-fos. While approaches 1 and 2 were effective in most cases, the paucity of parvalbumin in NTS (allowing its use as a negative marker of NTS) made it possible to unambiguously ascertain whether the electrode tip was in NTS and whether cfos-labeled neurons were in NTS. Our immunolabeling and cresyl violet staining procedures have been described in detail in our previous publications (Deng et al., 2007; Li et al., 2010, 2015). For the present two-color double labeling, sections were incubated in a rabbit primary antibody against c-fos at a 1:500 dilution (sc-52; Santa Cruz Biotechnology, Santa Cruz, CA, USA) overnight. After rinsing, the sections were incubated in donkey-anti rabbit secondary antibody (1:200; Jackson ImmunoResearch Laboratories, Inc., West Grove, PA, USA) for 1 h and rabbit peroxidase-anti-peroxidase (rabbit PAP; 1:1000; Jackson ImmunoResearch Laboratories, West Grove, PA, USA) for 1 h. Then, the c-fos signals were visualized by nickel-intensified DAB staining, yielding black nuclear labeling of c-fos+ neurons. Subsequently, the sections were incubated overnight in a mouse primary antibody against parvalbumin (1:5000; Sigma; P-3171). After rinsing, the sections were incubated in a donkey-anti-mouse secondary antibody and mouse PAP. Then the sections were processed using a standard brown DAB reaction, yielding brown parvalbumin neurons and processes. The H_2_O_2_ incubation time for the brown DAB reaction was 2 min (rather than the standard 10 min) so that the parvalbumin immunolabeling would be light and not interfere with detection of c-fos+ neurons. The sections were then rinsed, mounted on gelatin-coated slides, air-dried, dehydrated and coverslipped with Permount^®^ (Fisher Scientific, Pittsburgh, PA, USA). The sections were examined with a Olympus BH-2 microscope with standard transmitted light. Images were captured using a Spot Idea^TM^ camera and a Spot imaging software (Diagnostic Instruments, Inc., Sterling Heights, MI, USA). Note that only data from rats with confirmed, accurate NTS stimulation sites are presented here.

### Statistical Analysis

The effects of hypotension on c-fos expression in NTS neurons were evaluated by counts of labeled neurons in the control and hypotension conditions, and comparison by a *t*-test. We analyzed the effects of NTS stimulation on ChBF, ChBVol, ChBVel and ABP. For the time blocks prior to stimulation, we calculated the mean for ChBF, ChBVol, ChBVel and ABP, which served as the baseline level for these parameters against which to evaluate stimulation effects. For an analysis of the response to NTS activation, we compared the response during the 7 s of NTS stimulation to the response for that parameter in the 7 s prior to stimulation, using *t*-tests. For the time blocks during NTS activation in the mapping studies, we determined mean response for each rat, and then calculated group means. For the NPA studies, peak response, latency of the response (defined as 50% above basal) and time to peak during NTS activation were also determined. Data are presented separately for the NTS responses before NPA administration (pre-NPA) and after NPA administration (NPA), and the raw data analyzed by 2-way ANOVA with subsequent planned comparisons between groups by Fisher PLSD tests. Regression analysis was used to evaluate the contributions of ABP, ChBVol and ChBVel changes to ChBF changes. Group results are presented as mean ± SEM.

## Results

### Baroresponsive Subdivisions of NTS

**Figure 1:**
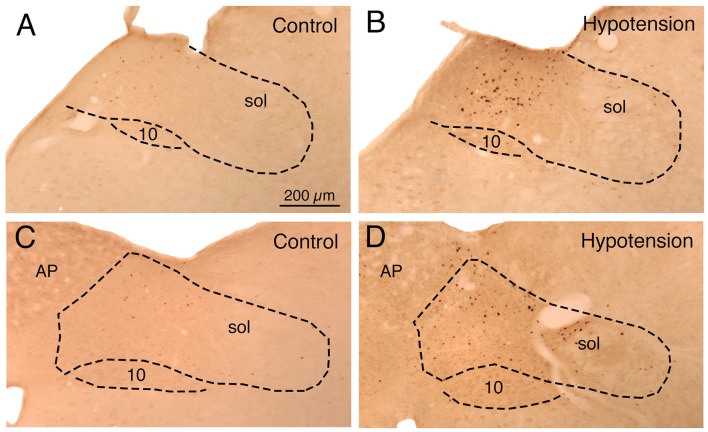
**Images showing the distribution of c-fos immunolabeling in: (1) nucleus of the solitary tract (NTS) just anterior to the area postrema (AP) in a sham hypotension rat (A) and a hypotensive rat (B); and (2) NTS at the level of AP in a sham hypotension rat (C) and a hypotensive rat (D).** The NTS is outlined, as is the vagal motor nucleus (10), and the location of the solitary tract (sol) is indicated. Note that hypotension induces c-fos in any neurons around and medial to the solitary tract. All images are at the same magnification.

**Figure 2:**
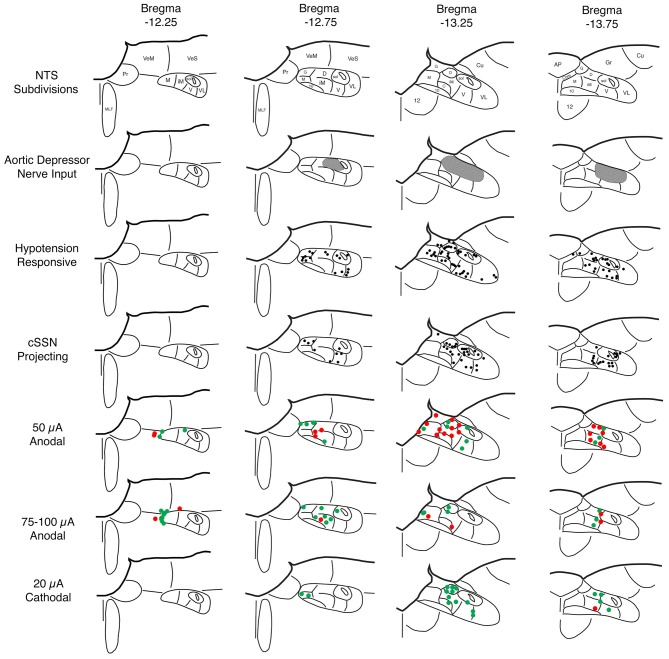
**Schematic illustration showing: (1) the NTS subdivisions (row 1); (2) the distribution of the aortic depressor nerve (ADN) input to superior salivatory nucleus (SSN) according to published studies by others (row 2; Ciriello, 1983; Housley et al., 1987; Altschuler et al., 1989); (3) the distribution of c-fos immunolabeling in NTS induced by hypotension as indicated in the present study and as consistent with prior reports by others (row 3; Rogers et al., 1993; Mayne et al., 1998); (4) the distribution of neurons in NTS that project to choroidal SSN (cSSN) as revealed from our prior studies involving intrachoroidal injection of pseudorabies virus (row 4; Li et al., 2015); (5) the distribution of sites within NTS yielding a significant choroidal blood flow (ChBF) increase (green) or no significant ChBF increase (red) in response to 50 μA anodal current pulses (row 5); (6) the distribution of sites within NTS yielding a significant ChBF increase (green) or no significant ChBF increase (red) in response to 70–100 μA anodal current pulses (row 6); and (7) the distribution of sites within NTS yielding a significant ChBF increase (green) or no significant ChBF increase (red) in response to 20 μA cathodal current pulses (row 7).** Note that hypotension responsive and cSSN neurons overlap one another, and both overlap but extend beyond the zone of ADN input. Thus, hypotension responsive and cSSN neurons either extend their dendrites into the ADN-receptive zone and/or receive input from AND-receptive neurons to account for the baroresponsiveness. The stimulation data indicate that ChBF increases can be driven by anodal stimulation in rostromedial NTS along the course of the axons traveling from NTS to cSSN and by cathodal stimulation within the region enriched in hypotension responsive and cSSN projecting neurons.

Sustained ABP 25–30% below basal was produced by 4–7 ml blood withdrawal to identify hypotensive responsive neurons in NTS, as assessed by c-fos immunolabeling. Counts of labeled neurons showed that the abundance of c-fos+ neurons following hypotension was tripled in baroreceptive NTS, notably in the dorsal, solitary, intermediate and ventral subdivisions of more caudal NTS (Figure 1). The increase was statistically significant (*p* < 0.043). The distribution of c-fos+ neurons in NTS after hypotension that we observed matches that described by others (Rogers et al., 1993; Mayne et al., 1998), and overlaps the terminal field within NTS of aortic and carotid sinus baroreceptor afferent input (Figure 2) via the vagus and glossopharyngeal nerves (Ciriello, 1983; Housley et al., 1987; Altschuler et al., 1989). Moreover, the distribution of c-fos+ neurons in NTS after hypotension matches the distribution of neurons within NTS that project to prechoroidal SSN (Figure 2) identified in our own work (Li et al., 2015). These findings are consistent with the notion that the NTS input to prechoroidal SSN is responsive to systemic ABP signals. Note that baroresponsive/SSN-projecting NTS neurons extend outside of the NTS zone receiving baroreceptive input, implying that neurons in baroreceptive NTS may project outside of this territory to neurons in baroresponsive/SSN-projecting NTS.

### Identification of NTS Sites Yielding ChBF Increases—50 μA Anodal Stimulation

**Figure 3:**
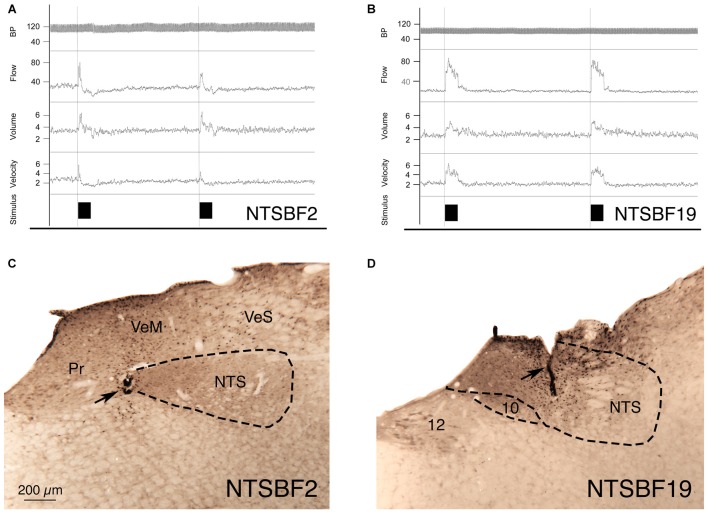
**Choroidal vasodilation with NTS stimulation from an illustrative case (NTSBF2) with 100 μA anodal stimulation (A,C), and an illustrative case (NTSBF19) with 20 μA cathodal stimulation (B,D). (A)** Shows recording traces for arterial blood pressure (ABP), ChBF, volume and velocity in response to 7 s anodal current pulse trains for NTSBF2 at the rostromedial NTS site shown by the arrow in **(C)**. During each NTS stimulation, ChBF, ChBVol, and choroidal blood velocity (ChBVel) increased for NTSBF2, but ABP did not. The effective stimulation site shown in this case is at the medial edge of the fiber tract traveling within NTS to cSSN. **(B)** Shows recording traces for ABP, ChBF, volume and velocity in response to 7 s cathodal current pulse trains for NTSBF19 at the more caudocentral NTS site shown by the arrow in **(D)**. During each NTS stimulation, ChBF, ChBVol, and ChBVel increased for NTSBF19, but ABP did not. The effective stimulation site shown in this case is within a part of NTS enriched in neurons projecting to cSSN. The magnification is the same in **(A,B)**.

**Figure 4:**
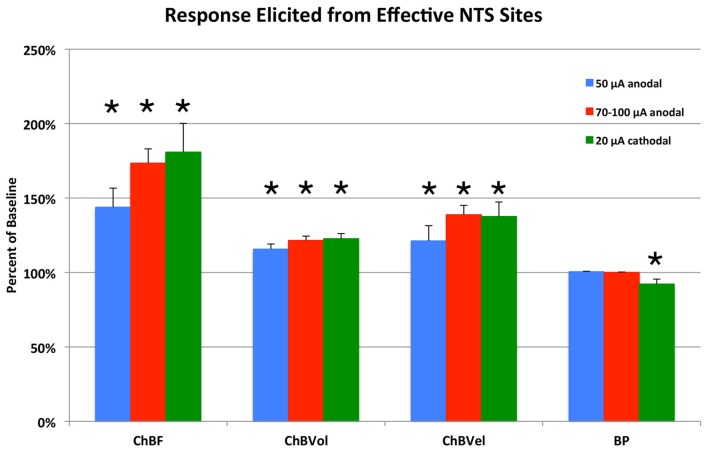
**Histogram showing the mean ChBF, ChBVol, ChBVel, and ABP responses to NTS stimulation at effective 50 μA anodal, 70–100 μA anodal, and 20 μA cathodal NTS stimulation sites, expressed as a percent of basal ChBF, ChBVol and ChBVel responses and ABP.** ChBF, ChBVol and ChBVel were significantly increased, but ABP was not at these sites. Asterisks indicate significantly different from baseline (100%). Error bars represent the SEM.

**Figure 5:**
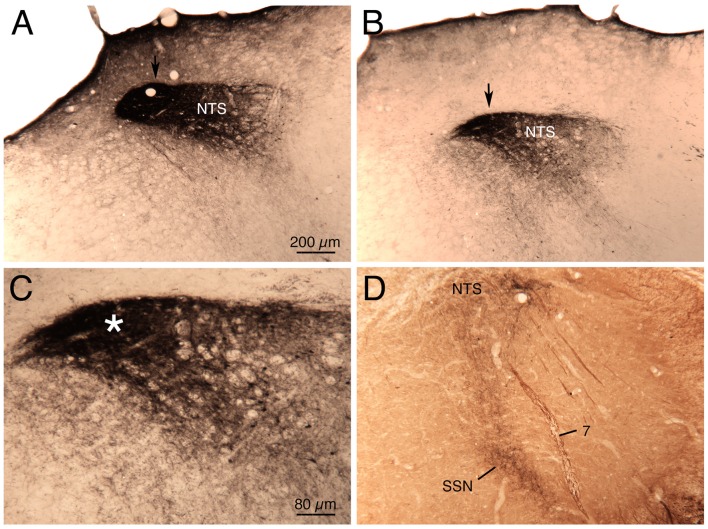
**Images showing the course of the axons from baroreceptive NTS coursing to prechoroidal SSN, as visualized in a previously described case (Li et al., 2010, 2015) in which biotinylated dextran amine was injected into NTS just anterior to the AP.** Images **(A,B,D)** show increasingly rostral levels through NTS, and the NTS axons are indicated by an arrow in **(A,B)**. Higher magnification view of the axons shown in image **(B)** is shown in image (**C**; asterisk). In image **(D)**, two-color DAB labeling has been performed to simultaneously detect the neurons (brown) of choroidal SSN for their enrichment in neuronal nitric oxide (NO) synthase, and the axons (black) coursing from NTS to the SSN. The magnification is the same in **(A,B,D)**.

Electrical activation of NTS with 50 μA anodal current pulses revealed loci that yielded ChBF increases by at least 10% above baseline (defined as effective sites), and others that did not (termed ineffective sites; Figures 2, 3). No differences were present in basal choroidal parameters or ABP for these sites (Table 1). The mean ChBF increases for the 17 effective sites with 50 μA anodal pulses were significantly elevated above baseline by 43.9% ± 12.7% (*p* = 0.0021), while there was no significant mean ChBF increase (1.0% ± 1.1%) for the 23 ineffective sites (*p* = 0.3502; Figures 2–4). Both effective and ineffective sites were observed in the baroreceptive and baroresponsive NTS subdivisions with 50 μA stimulation (Figure 2). Effective sites were most consistently seen in dorsomedial and rostral NTS, which coincides with the path of axons coursing from baroreceptive/baroresponsive NTS to prechoroidal SSN (Figure 5). The increases for the effective sites were accompanied by significant 15.7% ± 3.4% increases in ChBVol (*p* = 0.01 × 10^3^) and 21.3% ± 10.2% increases in ChBVel (*p* = 0.0462). As addressed in the “Discussion Section”, we interpret the volume increases for the effective sites to reflect vasodilation of choroidal vessels, and the velocity increases may largely reflect vasodilation of intraorbital choroidal feeder vessels. By contrast, no significant changes were seen for ChBVol (+1.0% ± 1.0%, *p* = 0.3509) or ChBVel (−0.3% ± 1.1%, *p* = 0.8100) with the ineffective 50 μA sites. Mean systemic ABP with effective 50 μA stimulation sites, by contrast to the choroidal effects, was only 0.5% ± 0.3% above basal, which was not significant (*p* = 0.0940; Figures 3, 4). Similarly, mean systemic ABP with ineffective 50 μA NTS sites was 1.3% ± 0.8% above basal (Figure 4), which was not significant (*p* = 0.1035). Thus, as ABP alterations caused by NTS activation were not significant, increased perfusion pressure due to increased ABP is unlikely to be a driver of the ChBF increases with 50 μA stimulation.

**Table 1:** **Basal choroidal parameters (ChBF, ChBVol and ChBVel) and basal arterial blood pressure (ABP) for the different sets of nucleus of the solitary tract (NTS) stimulation sites examined**.

Stimulation group	# of sites	Basal ChBF	Basal ChBVol	Basal ChBVel	Basal ABP
Effective 50 μA anodal sites	17	18.0 ± 1.21	2.98 ± 0.17	1.82 ± 0.07	104.7 ± 4.20
Ineffective 50 μA anodal sites	23	19.0 ± 1.17	2.94 ± 0.12	1.91 ± 0.08	101.9 ± 1.78
Effective 70–100 μA anodal sites	24	19.4 ± 1.32	3.30 ± 0.09	1.80 ± 0.09	102.2 ± 1.86
Ineffective 70–100 μA anodal sites	7	19.4 ± 2.20	3.40 ± 0.24	1.68 ± 0.10	95.7 ± 1.86
Effective −20 μA cathodal sites	18	15.0 ± 0.89	2.84 ± 0.08	1.60 ± 0.07	91.4 ± 5.31
Ineffective −20 μA cathodal sites	1	18.7	3.23	1.75	128.6
pre-NPA effective 100 μA anodal sites	8	21.1 ± 2.58	3.09 ± 0.15	2.06 ± 0.21	97.9 ± 5.76
With-NPA effective 100 μA anodal sites	8	16.8 ± 1.70	3.15 ± 0.11	1.81 ± 0.19	95.3 ± 5.78

### Identification of NTS Sites Yielding ChBF Increases—70 to 100 μA Anodal Stimulation

Similar results were seen with 70–100 μA current pulses, except that the mean increase above baseline for the 24 effective NTS sites was greater than for 50 μA pulses—73.5% ± 9.5% for 70–100 μA (*p* = 0.02 × 10^6^) vs. 43.9% ± 12.7% for 50 μA (Figures 2, 4). The effective sites were most commonly seen in the dorsal and intermediate subdivisions of NTS, and along the course of the NST axons traveling to prechoroidal SSN (Figure 2). Relatively fewer ineffective sites were observed with 70–100 μA anodal current pulses than with 50 μA pulses. No differences were present in basal choroidal parameters or ABP for these sites (Table 1). For ineffective sites (Figure 4), ChBF was not significantly changed from basal (+3.0% ± 1.8%, *p* = 0.2348). Neither stimulation of the effective NTS sites nor the ineffective sites yielded significant changes in mean ABP (Figure 4)—namely, +0.1% ± 0.2% for effective (*p* = 0.9045) and −0.1% ± 1.3% for ineffective (*p* = 0.8814). Thus, the ChBF increases with effective 70–100 μA NTS sites were not generated by effects of stimulation on ABP. Given that the ChBF increases for the 70–100 μA effective sites were accompanied by significant mean 21.5% ± 2.9% increases in ChBVol (*p* = 0.01 × 10^6^) and mean 38.8% ± 6.2% increases in ChBVel (*p* = 0.011 × 10^3^), the ChBF increases appear to be driven by vasodilation of choroidal vessels and in all likelihood intraorbital choroidal feeder vessels. The absence of a significant ChBF increase for ineffective 70–100 μA sites is consistent with the absence of significant changes in ChBVol (+1.8% ± 1.5%, *p* = 0.3115) or ChBVel (+2.5% ± 1.6%, *p* = 0.8814) for these sites.

### Identification of NTS Sites Yielding ChBF Increases—20 μA Cathodal Stimulation

Because anodal stimulation tended to drive ChBF increases most effectively from the axons traveling from NTS to prechoroidal SSN, we used cathodal stimulation of NTS in a second set of rats to provide better localization of the perikarya that drive ChBF increases from within NTS. We focused our penetrations on the subdivisions of NTS that receive baroreceptive input and are baroresponsive. Unlike with anodal stimulation, the 18 effective 20 μA cathodal NTS sites were localized primarily to baroreceptive/baroresponsive NTS—namely the dorsal, intermediate, ventral and solitary subdivisions (Figure 2). Only one ineffective site was found in this region with 20 μA cathodal stimulation (Figure 2). Basal choroidal parameters were similar for the effective sites to those for the one ineffective site, although ABP was much higher for the ineffective site (Table 1). A significant mean 80.9% ± 19.3% ChBF increase was observed for the effective sites (*p* = 0.00054), while for the ineffective site ChBF was only +2.6% above baseline (Figure 4). Similarly, ChBVol was +22.7% ± 3.4% above baseline (*p* = 0.03 × 10^4^) and ChBVel was +37.6% ± 9.7% above baseline (*p* = 0.0018). The ineffective site showed a large ChBVol decrease (−8.4%) but an equally large ChBVel increase (+10.7%), suggesting that this site had a mixed effect on ChBF. Although there was no ABP change with the ineffective site (+0.6%), a significant 7.7% ± 3.2% decrease in ABP was seen with 20 μA cathodal stimulation for effective NTS sites (*p* = 0.0295; Figure 4).

### Control Stimulation

To further evaluate the specificity of ChBF increases with NTS stimulation, we examined the effects of stimulation of cell groups around NTS. Activation of the hypoglossal nucleus ventromedial to NTS, the vagal motor nucleus along the lower medial border of NTS, or the cuneate or the medial and superior vestibular nuclei above NTS with anodal or cathodal stimulation commonly yielded eye, jaw or tongue movement, as well as variable effects on choroidal parameters and ABP. By contrast, such movements were rarely observed during NTS stimulation, and in those rare instances in which they were, the data were excluded, since the movements could produce artificial ChBF results.

### NTS Stimulation—Choroidal Blood Flow Dynamics

We used regression analysis to further assess the contributions of ABP, ChBVol and ChBVel to the observed ChBF increases with activation of effective NTS sites. For the 41 effective NTS sites observed with 50–100 μA anodal pulses, the ABP change (+0.32%) remained nonsignificant, and showed no significant positive correlation with ChBF, ChBVol, or ChBVel either prior to or during NTS stimulation. ChBF during 50–100 μA anodal NTS stimulation was much more highly correlated with ChBVel (*r* = 0.8998) than with ChBVol (*r* = 0.3198), although both correlations were significant. For the 18 effective 20 μA cathodal NTS sites, ChBVel was again significantly and highly correlated (*r* = 0.9845) with ChBF. In this case, however, ChBVol was also significantly and highly correlated with ChBF (*r* = 0.7279). Thus, intrachoroidal vasodilation was a greater contributor to ChBF increases with cathodal than anodal stimulation. Although a significant decline in ABP was observed during 20 μA cathodal NTS stimulation that yielded ChBF increases, the change in ABP was not significantly correlated with ChBF, ChBVol, or ChBVel. Thus, for cathodal stimulation as well as for anodal stimulation, any effects of NTS activation on ABP did not notably influence ChBF.

### NTS Stimulation—Pre-NPA Choroidal Blood Flow Effects

**Figure 6:**
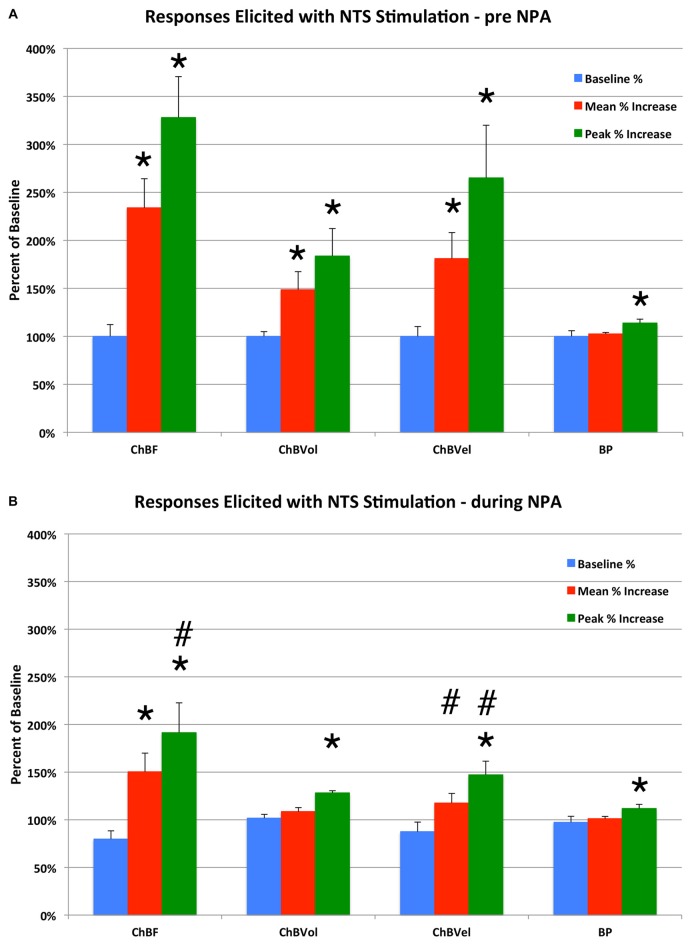
**Histogram showing the mean and peak ChBF, ChBVol and ChBVel responses and ABP responses to NTS stimulation at effective 100 μA anodal stimulation sites, expressed as a percent of basal ChBF, ChBVol and ChBVel responses and ABP, prior to N^ω^-propyl-l-arginine (NPA) administration (A) and after NPA administration (B).** Note that NTS stimulation prior to NPA yielded significant ChBF, ChBVol and ChBVel increases but had no effect on ABP, and that NPA attenuated the ChBF, ChBVol and ChBVel increases but had no effect on ABP. Asterisks indicate significantly different from baseline, and ampersands indicate significantly less than pre-NPA. Error bars represent the SEM.

**Figure 7:**
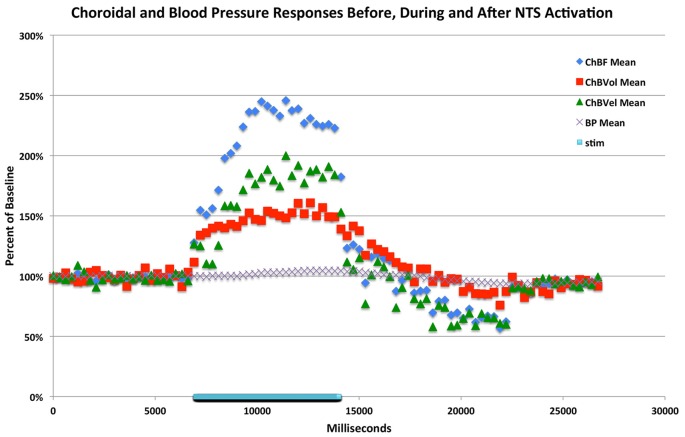
**Graph showing the time course of the mean ChBF, ChBVol, ChBVel and ABP responses to stimulation at effective anodal NTS sites (*n* = *x*).** The blue bar marks the stimulation period. Each data point is the mean for a 333 ms interval, and ChBF, ChBVol, ChBVel and ABP responses are all expressed as percent of basal. The rapid ChBF increases are driven by rapid increases in both ChBVel and volume.

In eight separate rats, we examined the effects of nNOS inhibition on NTS-elicited ChBF increases. For these studies, 100 μA anodal current pulses were delivered to NTS and yielded robust ChBF increases. The relative ChBF, ChBVol, ChBVel and ABP changes prior to, during and after stimulation were plotted for these rats to further examine the hemodynamics of the effect of electrical stimulation of NTS on ChBF, and as a baseline against which to compare NPA effects. NTS stimulation yielded significant mean increases above basal ChBF (+133.9% ± 30.3%, *p* = 0.002), basal ChBVol (+48.6% ± 18.8%, *p* = 0.0353), and basal ChBVel (+81.1% ± 27.1%, *p* = 0.0149), but no significant change in mean ABP from baseline (+2.6%, *p* = 0.7757) during the 7-s stimulation period (Figures 6, 7). The ChBF increase reached 50% above baseline by 0.94 s after current onset, and peaked at 2.99 s after stimulus onset. The ChBVol increased slightly more rapidly initially than did ChBVel, but their peaks were similar and occurred around 3.0 s after current onset. The NTS-stimulation-evoked increases in ChBF, ChBVol and ChBVel returned to baseline following stimulation offset, with a slight transient undershoot for ChBF and ChBVel but not ChBVol. NTS activation for these cases yielded a slight, gradual rise in ABP, resulting in a peak ABP (+15.9% ± 3.8%) at 4.45 s after onset of NTS stimulation onset that was, nonetheless, not significantly greater (*p* = 0.2596) than basal ABP. The ABP quickly returned to basal levels upon stimulation offset (Figure 7).

### Effect of nNOS Inhibition on ChBF Increase during NTS Stimulation

**Figure 8:**
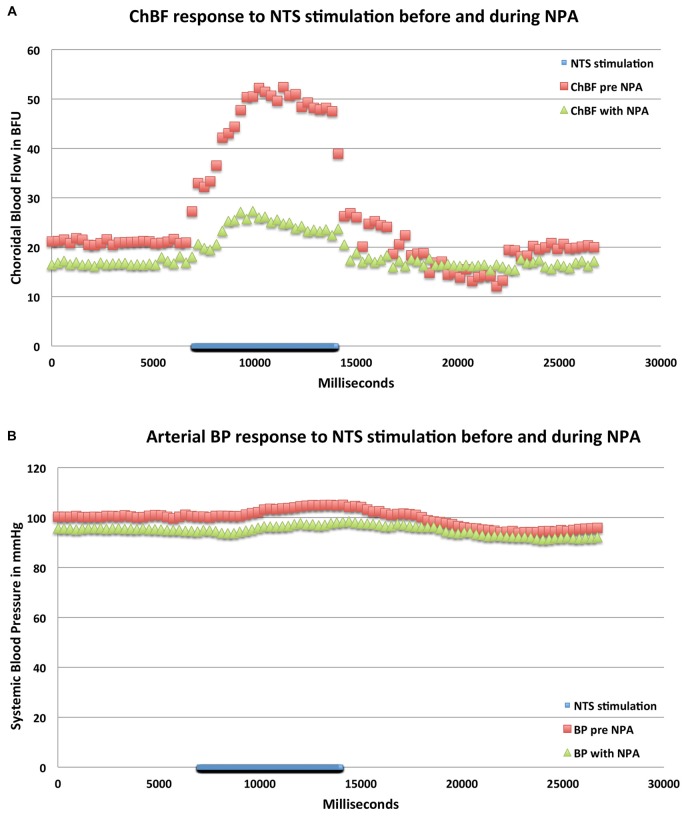
**Graphs showing the mean ChBF (A) and ABP (B) responses to stimulation at effective NTS sites prior to and after NPA administration (*n* = *x*).** The blue bar marks the stimulation period. Each data point is the mean for 333 ms. ChBF is expressed in relative blood flow units (BFU) and ABP in mm/Hg. Note that NPA significantly reduces the ChBF response to NTS activation, and has a consistent but insignificant depressive effect on ABP.

**Figure 9:**
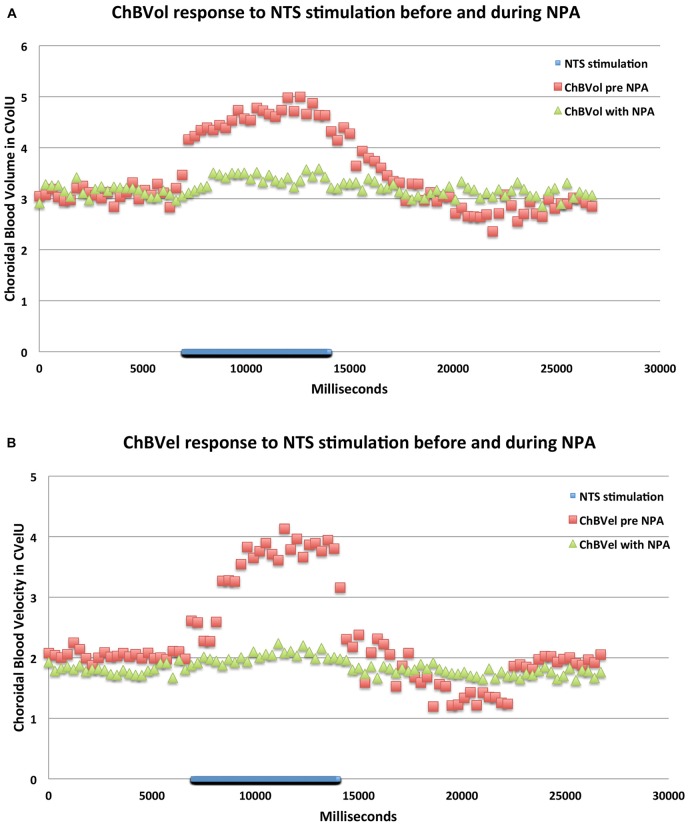
**Graphs showing the mean ChBVol (A) and ChBVel (B) responses to stimulation at effective NTS sites prior to and after NPA administration (*n* = *x*).** The blue bar marks the stimulation period. Each data point is the mean for 333 ms. ChBVol and ChBVel are expressed in relative units. Note that NPA significantly reduces both ChBVol and ChBVel responses to NTS activation.

Consistent with its action on nNOS but not endothelial nitric oxide synthase (eNOS), NPA had no significant effect on basal ABP (−2.5% ± 3.1%, *p* = 0.7549; Table 1; Figure 6). NPA also did not significantly alter mean basal ChBF (−16.3% ± 8.6%, *p* = 0.4836), although mean ChBF after NPA tended to be consistently lower over time (Table 1; Figures 6, 8). Basal ChBVol (+2.2% ± 2.4%, *p* = 0.9392) and ChBVel (−11.3% ± 6.9%, *p* = 0.6790) were also not significantly affected by NPA (Table 1). The effect of stimulation of NTS on mean and peak ChBF, ChBVol and/or ChBVel was, however, substantially reduced after NPA. For example, the mean ChBF increase with NPA was 48.9% ± 18.0% of that prior to NPA. Moreover, the mean ChBF during NTS stimulation after NPA treatment was no longer significantly above baseline (*p* = 0.2303). Additionally, the mean ChBF during NTS stimulation was significantly less after NPA than prior to NPA (*p* = 0.0008). Similarly, the peak ChBF with NPA was only 50.7% ± 19.0% of that during pre-NPA. Although the peak ChBF during NPA remained significantly above NPA baseline (*p* = 0.0245), the pre-NPA vs. NPA difference in peak ChBF increase with NTS stimulation was highly significant (*p* = 0.028 × 10^3^; Figures 6, 8). The ChBVol increase seemed comparably attenuated by NPA as the ChBVel increase during NTS stimulation (Figures 6, 9). For example, the mean ChBVol increase was only 34.5% ± 10.0% of pre-NPA, and the ChBVol after NPA was no longer significantly greater than basal ChBVol (*p* = 0.7227). Similarly, the mean ChBVel increase evoked by NTS activation after NPA administration was 28.8% ± 10.5% of pre-NPA, and was also no longer significantly greater than basal ChBVel after NPA (*p* = 0.7207). The response to NTS activation was also significantly less than observed prior to NPA (*p* = 0.0168). Thus, the effects of NPA on ChBF were driven by its inhibitory effects on both ChBVel and ChBVol. In general, NPA appeared to slightly slow the onset of choroidal responses and the time to peak (Figure 7).

## Discussion

This study shows that ChBF increases significantly upon activation of the baroresponsive part of NTS or its output fibers to prechoroidal SSN, and the increase in ChBF elicited by NTS activation is attenuated by the neuronal NOS inhibitor NPA. The ChBF increase appears to be mediated by vasodilation of orbital and choroidal blood vessels, but not by any effects of NTS activation on systemic BP. Our results indicate that NTS neurons projecting to choroidal SSN do mediate increases in ChBF. As these neurons tend to reside in the baroresponsive part of NTS, our results suggest a role of baroreceptive NTS in the well demonstrated BP-dependent regulation of ChBF (Kiel and Shepherd, 1992; Lovasik et al., 2003; Reiner et al., 2003, 2010b, 2011), which we refer to as baroregulation. These considerations are discussed in more detail below.

### NTS and ChBF Baroregulation Circuitry

The parasympathetic PPG innervates orbital vessels feeding the choroid (including the posterior ciliary arteries directly entering the choroid as well as the ophthalmic artery from which they arise), as well as choroidal vessels themselves (Stone et al., 1987; Cuthbertson et al., 1997, 2003; Jablonski et al., 2007; Reiner et al., 2012). The preganglionic neurons regulating the PPG reside in the SSN, the autonomic component of the facial motor complex in the hindbrain (Cuthbertson et al., 2003). In prior studies on the inputs to the SSN in rats, we found that a major input arose from the NTS of the medulla (Li et al., 2010), and in more recent studies we have demonstrated that the NTS regions that project directly to the ChBF-control neurons of SSN overlap the parts of NTS receiving cardiovascular afferents conveying ABP information (Figure 2; Li et al., 2015). The present study directly examined the subdivision-specific influence of NTS on the ipsilateral ChBF using LDF, and showed that activation of specific parts of NTS causes increases in ChBF. These regions include the part of NTS receiving direct baroreceptive input (ipsilateral interstitial and dorsal NTS subnuclei), the parts of NTS surrounding baroreceptive NTS that respond to hypotension, and the course of the axons projecting from NTS to choroidal SSN. Anodal current was more effective in stimulating the axons projecting from NTS to choroidal SSN, while cathodal stimulation was more effective in revealing the location of the NTS neurons driving ChBF increases. This outcome is in keeping with the reported relative efficacies of anodal vs. cathodal stimulation in activating axons vs. perikarya, respectively (Ranck, 1975; Bagshaw and Evans, 1976; Merrill et al., 2005; Lu et al., 2008). Our finding that activation of baroresponsive NTS increases ChBF in the ipsilateral eye is consistent with our prior evidence that this projection is glutamatergic (Li et al., 2015).

ChBVol was significantly increased during NTS stimulation-driven increases in ChBF. This is consistent with the vasodilation of choroidal vessels by activation of the PPG input to them as a result of NTS stimulation, which would be expected to manifest as an increase in ChBVol, i.e., ChBVol as measured by LDF. ChBVel was also significantly increased during NTS activation that increased ChBF. Choroidal vasodilation (increased vessel cross-sectional area) should, however, in principle decrease ChBVel, since blood velocity is inversely related to vessel cross-sectional area. Thus, it is unclear how ChBVel could increase with choroidal vasodilation. One possibility is that vasodilation occurs as well in the arteries feeding the choroid (ophthalmic and ciliary), increasing the volume of blood flow into the choroid and thereby increasing ChBVel. This would be expected as the PPG also innervates these feeder arteries, and activation of these fibers as well as direct application of the vasodilators they release dilate them (Wiencke et al., 1994; Nilsson, 1996; Toda et al., 1998; Ayajiki et al., 2000; Jarajapu et al., 2004; Overend et al., 2005; Overend and Martin, 2007; Reiner et al., 2012). Consistent with the impact of feeder vessel dilation on ChBF, ChBF is correlated with flow in orbital vessels that directly (posterior ciliary arteries) or indirectly (ophthalmic artery) supply the choroid, according to a number of studies (Dorner et al., 2003; Zion et al., 2007; Rechtman et al., 2007; DeoKule et al., 2009; Pemp et al., 2012; Novais et al., 2015). Thus, the increases in ChBVol and ChBVel driving the increases in ChBF during NTS stimulation may have been driven by vasodilation in different vascular beds. The increase in ChBVol is likely to reflect choroidal vessel vasodilation, while the increase in ChBVel may have been caused by dilation of orbital feeder vessels. Alternatively, it is also possible that the increase in both ChBVol and ChBVel that we observed entirely reflects complex dynamics of differential responses of choroidal arteries, veins and capillaries within the laser Doppler sampling volume to activation of the PPG input.

The PPG input to choroid and choroidal feeder vessels employs the vasodilators NO and VIP (Cuthbertson et al., 2003). As noted above, substantial evidence shows that both NO and VIP dilate orbital and choroidal vessels (Wiencke et al., 1994; Nilsson, 1996; Toda et al., 1998; Ayajiki et al., 2000; Jarajapu et al., 2004; Overend et al., 2005; Overend and Martin, 2007; Reiner et al., 2012). We found that NPA attenuated both the velocity-driven component and the volume-driven component of the ChBF increase. Given that NTS projects to the neurons of SSN that innervate PPG neurons innervating the entire geographic expanse of the choroid (Li et al., 2010) and that both NTS and SSN stimulation increase ChBF throughout choroid (Steinle et al., 2000; Fitzgerald et al. unpublished observations), our NPA data indicate that the ChBF increases elicited by NTS activation are likely to be mediated by activation of PPG neurons innervating choroid, and in all likelihood those innervating orbital vessels feeding choroid as well. The increase in ChBF with NTS activation that is not blocked by NPA inhibition of NO production may be mediated by VIP. Basal ChBF was not significantly diminished by NPA. It may be that basal ChBF is perhaps more dependent on VIP, and perhaps NO formed by endothelial NOS, than NO produced by PPG nerve terminals (Zagvazdin et al., 1996a,b). It should be noted, however, that in principle NPA could have blocked NO formation at the synapses between terminals of SSN prechoroidal neurons and their target PPG choroidal neurons, since SSN prechoroidal neurons have been observed by us to contain nNOS (Cuthbertson et al., 2003).

Our overall results suggest a role of baroresponsive NTS in the well demonstrated phenomenon of ChBF baroregulation, via its output to choroidal neurons of SSN. The NTS neurons projecting to choroidal SSN and those NTS sites yielding ipsilateral ChBF increases include both the parts of NTS receiving ABP-related cardiovascular afferents and surrounding parts of NTS that show responses to systemic hypotension. The baroresponsive neurons in the NTS territory surrounding baroreceptive NTS may receive their ABP information by means of input from baroreceptive NTS. It is unclear, however, how low BP signals to NTS from the aortic depressor nerve (ADN), which fires at a low rate during systemic hypotension (Zhang and Mifflin, 2000) could yield increased firing in the output to SSN, and ultimately to choroidal vasodilation. One possibility is that SSN-projecting NTS neurons receive innervation from inhibitory ADN-receptive NTS neurons, the latter of which may reside mainly in baroreceptive NTS and account for the SSN-projecting neurons found outside of baroreceptive NTS. During normal systemic ABP, the SSN-projecting NTS neurons would be inhibited by these ADN-receptive NTS neurons. When BP is low, ADN-receptive NTS neurons would fire at a low rate, with SSN-projecting NTS neurons then disinhibited. Other studies by us have supported this hypothesis (Li et al., 2016).

### NTS and Blood Pressure Regulation

The parts of NTS projecting to the SSN are known to receive ADN baroreceptive input via the vagus nerve and/or respond to systemic ABP fluctuation (Miura and Reis, 1972; Ciriello, 1983; Rogers et al., 1993; Zhang and Mifflin, 2000; Guyenet, 2006; Li et al., 2010), with increased BP increasing firing in the ADN and its target NTS neurons, and decreased ABP decreasing ADN firing and its target NTS neurons. The baroreceptive part of NTS is involved in regulation of systemic ABP by means of a multisynaptic output to sympathetic preganglionic neurons. In particular, baroreceptive NTS projects to GABAergic neurons of the caudal ventrolateral medulla (CVLM), which in turn project to excitatory neurons of the rostral ventrolateral medulla (RVLM) that project to sympathetic preganglionic neurons of the spinal cord (Schreihofer and Guyenet, 2003). Activation of baroreceptive NTS by high BP thus leads to activation of CVLM via NTS, leading to increased inhibition of RVLM, and diminished excitatory outflow from RVLM to sympathetic preganglionic neurons (Guyenet, 2006). This leads to reduced systemic vasoconstriction that serves to combat the heightened BP, and thereby mediates one part of the systemic baroreflex, together with decreased HR (Guyenet, 2006). Consistent with this, the present results showed reduced ABP with cathodal stimulation of baroresponsive NTS, as did some prior studies with electrical or chemical stimulation of baroreceptive NTS (Crill and Reis, 1968; de Jong et al., 1975; Talman et al., 1984).

### Functional and Eye Disease-Related Implications

The linkages between systemic and ocular circuitries, as in the case of NTS, reinforces the possibility that control of the two operates in parallel but in opposite directions—with the systemic sympathetic control serving to maintain systemic BP in the face of episodic declines in BP, and the parasympathetic control of ChBF serving to maintain high ChBF during such bouts of low systemic BP. Since the NTS also exerts a vasodilatory influence on cerebral blood flow (Nakai and Ogino, 1984; Agassandian et al., 2002, 2003), it may be that ocular and cerebral circulations are jointly regulated by the NTS→SSN→PPG circuit. Consistent with the protective role that cranial parasympathetic vascular regulation might play, severing PPG input to the cerebral vasculature intensifies the cerebral damage occurring with an ischemic event (Kano et al., 1991; Koketsu et al., 1992) and lesioning SSN leads to retinal pathology and functional impairment in rats (Reiner et al., 2010a). It may be that sympathetic innervation of the choroid and sympathetic innervation of peripheral blood vessels also work in opposite directions during ABP fluctuations, with (for example) high ABP leading to reduced sympathetic outflow to the periphery to mitigate the high ABP but enhanced sympathetic drive to the choroid to prevent overperfusion. Of particular note with regard to the eye, aortic baroreceptors provide the signal to NTS needed for activation of neurogenic choroidal baroregulation (Ciriello, 1983; Donoghue et al., 1985; Rogers et al., 1993), and baroreceptor function shows age-related decline, and is impaired by smoking, hypertension and diabetes (Brown et al., 1976; Fazan et al., 2001, 2006; do Carmo et al., 2007). The parasympathetic wing of baroregulation that mediates choroidal vasodilation via the NTS → SSN → PPG circuit during low BP may, thus, be impaired under these circumstances. This could help account for why choroidal baroregulation tends to be impaired by age, smoking, hypertension and diabetes (Movaffaghy et al., 2002; Polak et al., 2003; Wimpissinger et al., 2003; Reiner et al., 2011), and contribute to ChBF defects that harm the retina, and at least in part account for why these are all risk factors for age-related macular degeneration (Hageman et al., 2009).

## Author Contributions

CL, MECF, NDM and AR carried out research. CL, MECF and AR analyzed data. CL, MECF and AR wrote the manuscript.

## Funding

Supported by NIH-EY-05298 and The Methodist Hospitals Endowed Professorship in Neuroscience (AR), the Department of Ophthalmology at UTHSC (MECF) and the University of Tennessee Neuroscience Institute (CL).

## Conflict of Interest Statement

The authors declare that the research was conducted in the absence of any commercial or financial relationships that could be construed as a potential conflict of interest.

## Abbreviations

ABP: arterial blood pressure
AP: area postrema
BP: blood pressure
C: central subnucleus of NTS
ChBF: choroidal blood flow
ChBVol: choroidal blood volume
ChBVel: choroidal blood velocity
cSSN: choroidal subdivision of superior salivatory nucleus
Com: commissural nucleus of NTS
Cu: cuneate nucleus
CVLM: caudal ventrolateral medulla
D: dorsal nucleus of NTS
DAB: diaminobenzidine tetrahydrochloride
eNOS: endothelial nitric oxide synthase
G: gelatinous subnucleus of NTS
Gr: gracile nucleus
HR: heart rate
iM: intermediate subnucleus of NTS
M: medial subnucleus of NTS
MLF: Medial longitudinal fasciculus
nNOS: neuronal nitric oxide synthase
NO: nitric oxide
NPA: N^ω^-propyl-l-arginine
NTS: nucleus of solitary tract
Ox: blood oxygenation
Pr: nucleus prepositus
PPG: pterygopalatine ganglion
RVLM: rostral ventrolateral nucleus of medulla
sol: solitary tract and subnucleus of NTS
SSN: superior salivatory nucleus
stim: stimulation
V: ventral subnucleus of NTS
VeM: medial vestibular nucleus
VeS: superior vestibular nucleus
VIP: vasoactive intestinal polypeptide
VL: ventrolateral subnucleus of NTS
10: dorsal vagal motor nucleus
12: hypoglossal motor nucleus.
